# Editorial: Perception and Cognition: Interactions in the Aging Brain

**DOI:** 10.3389/fnagi.2016.00130

**Published:** 2016-06-08

**Authors:** Harriet A. Allen, Katherine L. Roberts

**Affiliations:** ^1^School of Psychology, University of NottinghamNottingham, UK; ^2^Department of Psychology, University of WarwickCoventry, UK

**Keywords:** aging, perception, cognition, vision, audition

Healthy aging can lead to declines in both perceptual and cognitive functions. Many of the studies in this Topic demonstrate such age-related declines, but also identify links between them. Encouragingly, these links suggest that improving perception could benefit cognition. In addition, while compensatory cognitive strategies were mainly unsuccessful in improving perception, cognitive training was effective under certain conditions.

## Common age-related decline

Cognitive and perceptual change may be linked because they are susceptible to the same age-related factors. In the present Topic, several studies suggest an age-related widening of tuning or a decrease of inhibition between representations. Both Chan et al. and McGovern et al. find that older adults are more likely to judge sound and lights presented asynchronously as synchronous. At the same time, Sapkota et al. find that older adults report more distractor items in a memory test. Pidgeon and Morcom also find that older adults are more susceptible to intrusions from distractor items, particularly if they are conceptually or perceptually similar to the target item. Similarly, Meinhardt-Injac et al. find that the unattended half of a face has a bigger influence on older (compared to younger) adults' judgments of face identity. These have some conceptual similarity with cognitive researchers' suggestions of age-related loss of focus, increased distractibility, and increased effects of similarity. For example, increased interference from distractors could be due to increased ambiguity in the way that target items are represented in the brain. In perceptual regions this could present as widening of tuning of receptors (Hua et al., 2006; Yu et al.; Betts et al., 2007). In higher-level areas this could present as less distinct category representations, leading to a higher reliance on “gist.”

The results of Hutchinson et al. and Komes et al. might be considered inconsistent with this broadening of tuning account. Hutchinson's finding of enhanced motion perception in older people could be interpreted as reflecting an age-related narrowing, rather than broadening, of tuning, while Komes' results indicate that poor memory is not associated with poor perceptual representations. In both cases, further research will help discriminate between a purely perceptual account and explanations that rely on attention and strategy changes. It therefore seems that while widening of tuning and more gist-based processing offer an explanation for a wide range of results, it is not yet a comprehensive explanation for all changes that occur with aging. It is not clear, for example, whether higher cognitive processes can offset the effects of perceptual tuning changes.

## From impaired perception to impaired cognition

Poor perception may lead to, or exacerbate, cognitive impairment (see Roberts and Allen for a review). As perceptual input gets harder to discriminate, more cognitive effort, or processes are required to decode the incoming signal. This may lead to worse performance by older adults because, effectively, the task they are performing is harder. Mishra et al. attempt to quantify this loss of cognitive resources through their Cognitive Spare Capacity Test.

When perceptual and cognitive deficits co-occur, cognitive skills may appear worse due to knock-on effects of poor perception on cognition. Thus, it might be tempting to ascribe a cognitive cause to what is, in fact, a perceptual deficit. Perceptual deficits can impact on cognition to the extent that a measure of (cognitively controlled) eye movements has potential to be used to diagnose eye disease (Crabb et al.). It is therefore important to fully account for perceptual impairment before concluding that there is a cognitive deficit, as was done in this Topic by Schoof and Rosen and Füllgrabe et al. In these studies, older adults were recruited who, unusually, had normal hearing sensitivity as measured by audiogram. Auditory temporal perception was also evaluated, as it is often impaired in older age and impacts on speech-in-noise perception. Even in these audiometrically-normal adults, the ability to understand speech in noise was predicted by a combination of temporal processing ability and cognitive performance (Füllgrabe et al.). Accounting for perception may therefore need to go beyond perceptual sensitivity to also consider suprathreshold processing (Allen et al., 2010; Füllgrabe et al.).

In the longer term, older adults with hearing impairment can show a faster rate of cognitive decline than those without (Lin et al., 2013). While this could be mediated by a separate factor, such as social isolation (Strawbridge et al., 2000), it may be that continual exposure to impoverished perceptual input leads to decrements in cognitive processes. A test of this idea is offered by Rönnberg et al., who discuss whether continued mismatch between perceptual input and long-term memory (LTM) representations could lead to less efficient LTM function. They find that even performance on a visually presented memory task is correlated with hearing loss, suggesting the impact of perceptual loss may be supramodal (Rönnberg et al.).

These studies suggest that the constant, and cumulative effort of coping with impaired perception could impact substantially on age-related cognitive decline.

## Cognitive improvements from training and perceptual interventions

In this Research Topic, while there was evidence for compensatory cognitive strategies, in most cases this led to different, rather than equivalent performance to younger adults. For example, reliance on gist led to increased false recognition (Pidgeon and Morcom), and reduced right-hemispheric dominance led to reduced pseudoneglect on a line bisection task (Benwell et al.). Komes et al. found that older adults who had more accurate memory for faces had a more bilateral electroencephalographic response than those with less accurate memory, presumably indicating some sort of cognitive compensatory process, but they still performed worse than younger adults.

In contrast, optimizing the perceptual input did, in some cases, lead to unimpaired cognition (e.g., Hutchinson et al.; Schoof and Rosen). This is demonstrated here by Rönnberg et al., who found that the impact of hearing loss on cognition could be mitigated via hearing aids. Although direct comparisons are limited by the difficulty of equating the complexity of perceptual and cognitive tasks, these results certainly put some limits on the extent of possible compensation mechanisms.

Older adults did show a benefit from cognitive training, particularly when that training was optimized for their needs. Casutt et al. improved older adults' on-road driving behavior and cognitive performance through training in a driving simulator. Meinhardt-Injac et al. found that older adults could benefit from feedback on a face-processing task when cognitive demands were low, although they were unable to benefit when cognitive demands were high.

## Methodological considerations

To clarify the relative contributions of perception and cognition, it may be tempting to use perceptual tests in one modality and cognitive tests in another (e.g., Rönnberg et al.; Schoof and Rosen; Füllgrabe et al.). Sometimes this is deliberate, so that cognitive measures will not be confounded by perceptual difficulties, but sometimes it is simply due to an established cognitive test being in a particular modality. It should be noted, though, that cognitive skills are not necessarily supramodal. Cognitive impairments such as neglect can arise in one modality but not another (e.g., Sinnett et al., 2007). Furthermore, some perceptual features are more critical to one modality than another (e.g., spatial location, Roberts et al., 2006, 2009).

It is also important to consider whether outcome measures are optimized to detect age-related effects. Gordon et al. report that functional magnetic resonance imaging measures of the peak and spread of activation are separately informative about older adults' performance on a cognitive task. During performance on a Sternberg task, some age-related effects were only apparent when looking at measures of spread. Komes et al. also found differences in the distribution of (EEG) activation in older adults with good vs. poor memory.

## Conclusions

The link between perception and cognition is not well understood. It is possible that perception and cognition are affected by the same, superordinate cause. How much of age-related change can be explained by gradual widening of tuning or categories is a promising and interesting route for further work. This could also help to improve older adults' performance on cognitive tasks. For example, where older adults show a broadening of representations or increased reliance on gist, cognitive performance could be improved by providing more distinctive or spatially-separated stimuli (Pidgeon and Morcom; Sapkota et al.).

While broadening of tuning appears to have multimodal effects, other interactions between perception and cognition appear to differ between modalities. Impaired perception may put a load on cognition, perhaps eventually leading to capacity loss. It is interesting to note, however, that these studies are predominantly from the auditory domain. It remains to be seen whether this generalizes to visual stimuli.

## Author contributions

All authors listed, have made substantial, direct and intellectual contribution to the work, and approved it for publication.

### Conflict of interest statement

The authors declare that the research was conducted in the absence of any commercial or financial relationships that could be construed as a potential conflict of interest.

## References

[B1] Allen H. A. Hutchinson C. V. Ledgway T. Gayle P. (2010). The role of contrast sensitivity in global motion processing deficits in the elderly. J. Vis. 10:15. 10.1167/10.10.15 20884480

[B2] Betts L. R. Sekuler A. B. Bennet P. J. (2007). The effects of aging on orientation discrimination. Vision Res. 47, 1769–1780. 10.1016/j.visres.2007.02.016 17466355

[B3] Hua T. Li X. He L. Zhou Y. Wang Y. Leventhal A. G. (2006). Functional degradation of visual cortical cells in old cats. Neurobiol. Aging 27, 155–162. 10.1016/j.neurobiolaging.2004.11.012 16298251

[B4] Lin F. R. Yaffe K. Xia J. Xue Q.-L. Harris T. B. Purchase-Helzner E.. (2013). Hearing loss and cognitive decline in older adults. JAMA Intern. Med. 173, 293–299. 10.1001/jamainternmed.2013.1868 23337978 PMC3869227

[B5] Roberts K. L. Summerfield A. Q. Hall D. A. (2006). Presentation modality influences behavioral measures of alerting, orienting, and executive control. J. Int. Neuropsychol. Soc. 12, 485–492. 10.1017/S1355617706060620 16981600

[B6] Roberts K. L. Summerfield A. Q. Hall D. A. (2009). Covert auditory spatial orienting: An evaluation of the spatial relevance hypothesis. J. Exp. Psychol. Hum. Percept. Perform. 35, 1178–1191. 10.1037/a0014249 19653757

[B7] Sinnett S. Montserrat J. Rafal R. Azanon E. Soto-Faraco S. (2007). A dissociation between visual and auditory hemi-inattention: evidence from temporal order judgements. Neuropsychologia 45, 552–560. 10.1016/j.neuropsychologia.2006.03.006 16690089

[B8] Strawbridge W. J. Wallhagen M. I. Shema S. J. Kaplan G. A. (2000). Negative consequences of hearing impairment in old age: a longitudinal analysis. Gerontologist 40, 320–326. 10.1016/j.neuropsychologia.2006.03.006 10853526

